# Mentalizing in the margins: a pilot study of group-based MBT-TF for underserved neurodiverse late adolescents

**DOI:** 10.3389/fpsyt.2026.1743200

**Published:** 2026-04-28

**Authors:** Dawn. L. Bales, Mirjam E. J. Kouijzer

**Affiliations:** 1 MBT-Expertise, MBT-Expertise Behandelcentrum, Breda, Netherlands; 2 GGz Breburg, Breda, Netherlands

**Keywords:** adolescence, group therapy, MBT-TF, mentalization based treatment, neurodiversity, trauma

## Abstract

**Introduction:**

Complex trauma can severely impair mentalizing capacities and epistemic trust, leading to pervasive difficulties in self-concept, emotion regulation, and relationships. Late adolescents with co-occurring complex posttraumatic stress disorder, personality dysfunction, and features of autism spectrum disorder represent a particularly underserved and clinically complex group. This pilot study explored the feasibility and perceived impact of a group-based adaptation of Mentalization-Based Treatment for Trauma (MBT-TF) tailored to the developmental and neurodiverse needs of this population.

**Methods:**

Four late adolescents aged 18–23 participated in a six-month adapted MBT-TF module delivered within a specialized mental health program. The intervention incorporated structural modifications to enhance predictability, visual support, and emotional safety. A mixed-methods design combined pre- and post-treatment self-report questionnaires with qualitative interviews assessing treatment engagement, acceptability, and perceived change.

**Results:**

Attendance and engagement were high across all participants. Thematic analysis identified improvements in self-image, emotion regulation, reflective functioning, and relational trust, supported by the structured and predictable group format. Participants reported increased self-compassion, reduced shame, and a sense of belonging within the neurodiverse peer group. Quantitative data showed favorable trends in reflective functioning, interpersonal problems, dissociation, and posttraumatic stress symptoms, with several participants demonstrating clinically meaningful change.

**Discussion and conclusion:**

Findings suggest that group-based MBT-TF is feasible, acceptable, and potentially beneficial for neurodiverse late adolescents with complex trauma and personality difficulties. Structural adaptations emphasizing predictability, collaboration, and visual scaffolding appear to enhance epistemic trust and engagement. Future research should evaluate these preliminary findings in larger samples to further refine trauma-focused mentalization-based approaches for neurodiverse populations.

## Introduction

Complex trauma, particularly when rooted in prolonged interpersonal adversity, undermines epistemic trust and impairs mentalizing capacities, which in turn disrupts the development of self, emotion regulation, and relational functioning. Late adolescents presenting with a combination of complex trauma, personality pathology, and neurodiverse traits represent a particularly high-risk and clinically complex population. These individuals frequently fall between existing treatment modalities, as standard trauma-focused or personality-focused interventions often exclude this group or fail to adequately address their multifaceted needs.

Mentalization-Based Treatment (MBT) is an evidence-based psychotherapeutic approach designed to enhance epistemic trust and mentalizing capacities, particularly in individuals with personality disorders. To optimize treatment outcomes in patients with significant trauma histories, a trauma-focused adaptation—Mentalization-Based Treatment for Trauma (MBT-TF)—was developed for individuals with complex trauma and co-occurring borderline personality pathology (1, 2).

MBT-TF is a structured, group-based intervention that integrates trauma processing within a mentalizing framework. It follows a phased treatment model: (1) stabilization and psychoeducation, (2) trauma processing, and (3) mourning and integration. MBT-TF is based on the idea that trauma—especially attachment trauma—makes it difficult for people to process their experiences in connection with others. When stress and attachment systems are affected, mentalizing capacity and epistemic trust are weakened. Recovery depends on enhancing epistemic trust and making sense of experiences together. To support this, MBT-TF offers a structured group setting where participants work on a collaborative trauma formulation, learn to connect bodily sensations with emotions (embodied mentalizing), and develop a shared understanding of their difficulties and traumatic experiences in a safe and predictable environment. Through these mechanisms, MBT-TF aims to reduce psychological isolation, shame, and social vigilance, while fostering epistemic trust and relational recalibration (2).

To date, MBT-TF has only been applied to adult populations without ASD traits. Its feasibility in younger populations—particularly those with neurodevelopmental vulnerabilities—remains unexplored. However, feasibility studies have shown that MBT-TF can be successfully implemented in group settings for adults with complex trauma (1). Late adolescents (aged 18–23) are in a developmentally sensitive phase, navigating identity formation, autonomy, and relational complexity. When presenting with co-occurring C-PTSD, personality dysfunction, and ASD features, the clinical picture becomes especially intricate.

Although autism spectrum disorder (ASD) is traditionally conceptualized as a neurodevelopmental condition with a strong genetic basis, recent research increasingly highlights the role of early relational experiences—particularly attachment disruptions—in shaping socio-emotional development in individuals with ASD (3, 4). Children and adolescents with ASD are disproportionately exposed to adverse childhood experiences (ACEs), including neglect, abuse, and relational trauma, which may compound existing vulnerabilities in emotion regulation and mentalizing (5). Moreover, the bidirectional nature of attachment relationships suggests that ASD-related social and emotional characteristics may challenge caregiver responsiveness, potentially leading to reciprocal disturbances in attachment formation (4, 6).

Importantly, there is growing concern that trauma-related impairments in mentalizing—especially when rooted in attachment trauma—may sometimes be misinterpreted as core features of ASD. In clinical practice, individuals with reduced capacity for social understanding, emotional reciprocity, and perspective-taking may be mistakenly diagnosed with ASD, particularly when the etiology of these difficulties is unclear (7, 8). Several studies have documented cases in which individuals with histories of trauma or personality pathology received an ASD-diagnosis later in life, often after years of misdiagnosis with other psychiatric conditions such as psychosis, personality disorders, or intellectual disability (7, 9). There are also many known cases in which the misdiagnosis went the other way around. The diagnostic overlap underscores the need for careful differential assessment, especially in populations with complex trauma histories and impaired mentalizing capacities.

Given the multiple and increasingly recognized links between attachment trauma and autism spectrum traits—ranging from developmental vulnerability to diagnostic complexity—there is a clear rationale for exploring trauma-focused interventions in this population. MBT-TF, with its emphasis on restoring epistemic trust, enhancing mentalizing, and processing trauma within a structured relational context, offers a theoretically grounded and clinically promising framework.

The decision to apply MBT-TF in this pilot study was based on the hypothesis that its phased, trauma-informed, and group-based format could address core vulnerabilities in both trauma and neurodevelopmental domains. However, given the specific cognitive and emotional needs of late adolescents with ASD traits—such as a need for extra predictability, concrete and visual structure, and support in navigating relational dynamics—adaptations to the standard MBT-TF protocol are likely necessary to ensure accessibility, safety, and therapeutic engagement. This pilot study therefore aimed to explore the feasibility of MBT-TF in this population, hypothesizing that its structured, relational, and mentalizing-based approach could offer meaningful support for individuals with co-occurring C-PTSD, personality dysfunction, and ASD features.

This pilot study is the first to explore the feasibility of an adapted MBT-TF module as an add-on to a adapted MBT-A program for late adolescents with co-occurring C-PTSD, personality dysfunction, and ASD features. The module was slightly modified to better align with the developmental stage and clinical needs of this population. Quantitative and qualitative data were collected to assess treatment acceptability, engagement, perceived safety, and preliminary indicators of clinical change.

## Methods

### Participants

Four late adolescents between the ages of 18 and 23 participated in the study. Three identified as female, and one was assigned female at birth but identified as male at the time of the study. All participants had a history of long-term involvement in mental health care and had received treatment since early childhood. Their clinical profiles were marked by a high degree of complexity, including multiple and shifting psychiatric diagnoses over time, such as autism spectrum disorder and ADHD, depressive disorders, Tourette syndrome, PTSD, eating disorders, conversion disorder, gender dysphoria, and several personality disorders. At the start of the group, none of the participants were engaged in education or employment, and three were living in supported psychiatric housing facilities rather than with their families. All had experienced one or more psychiatric crisis admissions in the past. Each had also previously discontinued other treatment programs, such as ‘regular’ MBT-A, due to the severity of their difficulties and limited capacity to benefit from standard interventions.

### Procedure

All participants were already in treatment at a specialized mental health care facility in the Netherlands at the time of inclusion. For these late adolescents, trauma-focused Mentalization-Based Treatment (MBT-TF) was offered because other available treatment programs had proven insufficient or unsuitable, and MBT-TF was considered the most appropriate next step. All participants were being treated in an adapted MBT-A program, had developed an individual crisis plan, and expressed willingness to engage in the group treatment. Participants provided written informed consent prior to participation. They were informed that participation was voluntary and that they could withdraw at any time without consequences for their treatment. The study was reviewed and approved by the institutional Scientific Research Ethics Committee of the mental health organization in which the research was conducted. Standardized self-report questionnaires were administered before and after the group and following completion of the program each participant also took part in a semi-structured interview to explore their experiences with the treatment.

### Treatment duration, attendance, and drop-out

The standard MBT-TF protocol for adults consists of a nine-month program that includes weekly group sessions of 1.5 hours in combination with weekly individual MBT sessions. For the present pilot, the trauma-focused module was implemented as an add-on requiring several structural adjustments to meet the neurodiverse late-adolescent population’s needs. Specifically, the duration of group sessions was shortened from 90 minutes to 75 minutes to better align with participants’ cognitive and emotional processing capacities. The adapted protocol comprised 23 weekly sessions delivered over a six-month period.

Attendance across the group was exceptionally high. There were no drop-outs and all four participants completed the full module. Only two participants missed one session each (one due to a pre-planned vacation, one due to acute illness). One additional participant attended one session online because of illness; this was interpreted by the clinical team as an indicator of strong motivation and treatment commitment. Overall, these data demonstrate strong feasibility, high acceptability, and robust adherence despite participants’ histories of treatment discontinuation and complex clinical presentations.

### Treatment program adaptions

The trauma-focused Mentalization-Based Treatment (MBT-TF) add-on module was delivered by two therapists trained in Mentalization-Based Treatment and followed the established MBT-TF manual. Because the standard MBT-TF protocol was originally developed for adults with more typical cognitive profiles, several adaptations were made to meet the developmental and neurodiverse needs of these late adolescents. These adaptations were informed by clinical experience and grounded in established scientific insights, and were designed to enhance predictability, concreteness, and accessibility within the group process.

To optimize emotional safety, predictability, and relational attunement, we deliberately chose for a small (4 participants) and neurodiverse-homogeneous group format. A smaller group size was chosen to reduce social and sensory overload, limit the number of simultaneous interpersonal cues, and create a more manageable relational context for participants. In addition, assembling a group with similar neurocognitive styles and comparable levels of complexity—including longstanding difficulties across educational, social, and treatment domains—helped establish shared expectations and lowered the interpersonal demands that often arise in more heterogeneous groups. Participants were placed together in a small, neurodiverse group to foster mutual understanding and reduce social stress—an approach supported by recent findings suggesting that autistic individuals often experience more effective social interaction within homogeneous peer groups (10). This choice is also supported by research on the double empathy problem (11), which demonstrates that social misunderstandings between individuals with differing neurocognitive profiles tend to be reciprocal rather than one-sided, and that interaction quality improves substantially when communication partners share similar experiential and processing frameworks. Such findings show that neurodiverse-homogeneous groups often facilitate greater mutual understanding, smoother interaction, and increased relational safety compared to mixed-neurotype groups. Within this framework, the small, homogeneous group was intended to reduce interpersonal mismatch effects, support engagement, and create conditions in which mentalizing processes could unfold with less communicative strain.

To accommodate participants’ cognitive and emotional processing capacities, all sessions across phases were shortened from 90 to 75 minutes while maintaining therapeutic depth and continuity. Participants also expressed a strong need for structural consistency. To meet this need, fixed seating arrangements, a consistent order for check-in and closing rounds, and standardized formats for reviewing sessions were introduced. These elements contributed to emotional safety, predictability, and reduced cognitive load.

In Phase 1, psychoeducation was presented in a more concrete and visually structured manner. Each participant received a personalized visual schedule outlining weekly themes and activities, which remained visible throughout the module to support orientation and reduce anxiety. Participants additionally created individualized flip-charts containing their trauma-related re-enactment cycle, patterns of hyper- and hypoarousal, and concrete self-regulation strategies. These visual tools were used actively during sessions to support mentalizing and co-regulation. Integration of these elements extended Phase 1 by three additional sessions, allowing sufficient time for stabilization and shared understanding.

In Phase 2, trauma narrative sessions were adapted to include peer-generated questions. Group members were invited to ask questions during narrative sharing, which enriched the mentalizing process and provided perspectives beyond those anticipated by therapists. To allow participants more time for personal reflection, the traditional “take-home message” was replaced by a structured self-report format in which participants documented their insights in writing after each session. This format was co-developed with participants, who were explicitly invited to reflect on what types of questions and formats would support their trauma processing. To foster integration and cohesion, review sessions were scheduled after every two trauma processing sessions, revisiting group values and norms and reflecting on the ongoing use of each participant’s flip-chart.

Phase 3 was shortened, as most participants continued treatment within the adapted MBT-A program following completion of the group. Across all phases, sessions retained a consistent structure including check-in, exploration of weekly themes, experiential or reflective exercises, and a closing round. The structured yet flexible design fostered emotional safety, encouraged mentalizing within interpersonal contexts, and supported the integration of trauma-related experiences.

### Instruments

All participants completed a set of self-report questionnaires both before and after the group treatment. Participants completed the Inventory of Interpersonal Problems (IIP), the Dissociative Experiences Scale–II (DES-II), the PTSD Checklist for DSM-5 (PCL-5), the Reflective Functioning Questionnaire (RFQ), and the Childhood Trauma Questionnaire (CTQ). These instruments were selected to assess a range of relevant domains, including interpersonal functioning, dissociation, post-traumatic stress symptoms, reflective functioning, and trauma history.

The *Inventory of Interpersonal Problems (IIP-32)* (12) assesses interpersonal difficulties across eight domains, i.e. Dominating, Intrusive, Overly Nurturing, Exploitable, Nonassertive, Socially Inhibited, Cold, and Vindictive. Participants rate how distressing each item is on a 5-point Likert scale ranging from 0 (*not at all*) to 4 (*extremely*). The outcome variable in this study is the total interpersonal distress, calculated by adding the scores on all interpersonal problem domains.

The *Dissociative Experiences Scale–II (DES-II)* (13) measures the frequency of dissociative experiences. Items are rated on a visual analog scale from 0% to 100%, indicating the percentage of time each experience occurs. The outcome is an average total score, with higher scores indicating more frequent dissociative symptoms.

The *PTSD Checklist for DSM-5 (PCL-5)* (14) assesses the severity of PTSD symptoms over the past month, based on DSM-5 criteria. It consists of 20 items rated on a 5-point Likert scale from 0 (*not at all*) to 4 (*extremely*). The total severity score is used as the outcome variable.

The *Reflective Functioning Questionnaire (RFQ)* (15) assesses individuals’ capacity to understand mental states in oneself and others. It contains two subscales: *Certainty about Mental States* and *Uncertainty about Mental States*, with items rated on a 7-point Likert scale from 1 (*completely disagree*) to 7 (*completely agree*). The total score on both subschales was used as outcome variable.

The *Childhood Trauma Questionnaire (CTQ)* (16) retrospectively assesses experiences of abuse and neglect during childhood. Items are rated on a 5-point Likert scale from 1 (*never true*) to 5 (*very often true*). It yields subscale scores for emotional, physical, and sexual abuse, and emotional and physical neglect, as well as a total score, which is used as outcome variable in this study.

In addition to the standardized questionnaires, all participants took part in an individual semi-structured interview within one month after completing the group. The interviews were conducted at the mental health care facility, lasted approximately one hour, and were aimed at exploring participants’ subjective experiences with MBT-TF. Topics covered included the reasons for initiating the module, treatment goals and expectations, perceived changes in self and relationships, the process of sharing and hearing traumatic memories, and the added value of the module compared to regular treatment. Participants were also asked about the impact of the module on their individual and systemic therapy, aspects they found helpful or unhelpful, suggestions for improvement, and what they considered most valuable about the program.

### Data analysis

A mixed-methods approach was used to analyze the data. Quantitative analyses were conducted on the questionnaire data collected before and after the group treatment. Descriptive statistics were calculated for each outcome measure, and pre-post changes were explored using within-subject comparisons to examine trends in symptom reduction and psychological functioning. Given the small sample size (n = 4), statistical analyses were exploratory in nature and primarily descriptive. In addition, Reliable Change Indices (RCIs) were calculated for individual participants to evaluate whether observed changes exceeded measurement error.

In addition, a qualitative analysis was conducted on the post-treatment interviews to gain deeper insight into participants’ subjective experiences of the group process and perceived effects. Interviews were audio-recorded, transcribed verbatim, and analyzed using reflexive thematic analysis following the six-phase framework described by Braun and Clarke (17).

The analysis was conducted by the second author, who was independent from the clinical team and not involved in the development, adaptation, or delivery of the TF-MBT module. This researcher also did not conduct the interviews and had no prior therapeutic or clinical relationship with the participants. This independence was intended to minimize potential allegiance effects and enhance interpretive neutrality.

The analytic process began with repeated engagement with the transcripts, followed by inductive coding across the dataset. Initial codes captured both positive and challenging experiences, including ambivalence, uncertainty, and perceived limitations of the intervention. Codes were then iteratively reviewed, refined, and grouped into candidate themes, which were further developed through repeated comparison with the original data to ensure coherence, internal consistency, and clear differentiation between themes.

## Results

### Qualitative analyses

Thematic analysis of the post-treatment interviews revealed five overarching themes that captured participants’ experiences and perceived changes throughout the trauma-focused MBT group. These themes were: (1) self-image and self-worth, (2) emotional self-care and regulation, (3) insight and reflection, (4) relationships and social interaction, and (5) boundaries, safety, and structure.

1. Self-image and self-worth.

Participants described how the group helped them develop a more realistic and compassionate view of themselves. Several initially struggled to recognize the impact of early neglect, or held a distorted self-image shaped by trauma and invalidation. Through recognition, validation, and shared experiences in the group, they began to experience a shift: “I realized I wasn’t exaggerating — it really was neglect.” Subthemes included feeling more authentic (“I can be myself”), increased self-worth (“I matter”), and feeling taken seriously and understood by others. For some, this marked the beginning of developing a coherent and stable sense of self.

2. Emotional self-care and regulation.

Participants reported increased capacity to understand and manage emotional experiences. The group helped them recognize how harsh they had been toward themselves and begin to cultivate greater self-compassion and emotional tolerance. Several noted a reduction in feelings of self-disgust and shame, and a new ability to identify and cope with tension or emotional triggers. A recurrent experience was the realization that the trauma was not their fault, often accompanied by a sense of relief and self-forgiveness. “I always thought it was me — but now I see it differently.”

3. Insight and reflection.

The group facilitated deeper understanding of personal experiences, including the origins and context of their difficulties. Participants described learning to take a reflective stance toward their thoughts, emotions, and behaviors — a capacity often associated with mentalizing. Through psychoeducation and group discussion, they gained tools to step back from overwhelming experiences and consider alternative perspectives. This helped them place their trauma within a broader narrative and reduce self-blame. “Now I can look at things from more than one angle.”

4. Relationships and social interaction.

Many participants reported positive changes in how they related to others. The group experience fostered trust, openness, and a growing belief that others could be safe and accepting. Feeling seen and taken seriously by fellow group members contributed to a sense of connection and belonging. Several participants also described greater confidence and assertiveness in relationships outside the group, and an ability to apply interpersonal insights in daily life. “For the first time, I dared to show something real.”

5. Boundaries, safety, and structure.

The structured and predictable format of the group was frequently mentioned as essential to participants’ sense of emotional safety. This stability enabled them to reflect on, discuss, and process painful and challenging material. Participants reported feeling increasingly able to express their needs and set boundaries — both within and outside the group. The group was described as a safe environment in which new relational experiences could emerge. “Because I knew what to expect, I could finally let my guard down a little.”

Across interviews, participants described feeling consistently engaged and emotionally invested in the group, reporting a level of depth, openness, and continuity that contrasted with their previous difficulties staying committed in treatment.

### Quantitative analysis

Descriptive analyses of the questionnaire data (n = 4) indicated mixed but generally favorable trends across several domains of functioning. Given the small sample size, results should be interpreted as exploratory. Pre and post scores of the participants on all questionnaires can be found in **Appendix 1**.

On the Childhood Trauma Questionnaire (CTQ), scores remained largely stable, as expected given that the measure captures retrospective reports of childhood adversity.

The Reflective Functioning Questionnaire (RFQ) showed a modest overall increase in reflective functioning from pre- to post-treatment. However, due to missing data for one participant and the lack of established clinical cut-offs, interpretation remains tentative.

In terms of interpersonal functioning, as measured with the Inventory of Interpersonal Problems (IIP), all participants showed improvement over the course of the intervention. One participant demonstrated a clinically significant change, as determined using the Reliable Change Index (RCI).

According to the Dissociative Experiences Scale (DES-II), one participant experienced a significant decrease in dissociative symptoms. One participant, however, reported an increase in dissociative experiences.

Finally, the PTSD Checklist for DSM-5 (PCL-5) suggested a general decline in PTSD symptom severity across the group, with one participant showing reliable improvement, and one a moderate decrease in symptoms and one showing a slight increase in symptoms.

## Discussion

This pilot study explored the feasibility and potential benefits of group-based Mentalization-Based Treatment for Trauma (MBT-TF) in a small sample of underserved neurodiverse adolescents with complex trauma histories and personality dysfunction. Despite individual variability, both qualitative and quantitative data suggest that MBT-TF may offer meaningful therapeutic gains in domains such as reflective functioning, interpersonal relationships, dissociation, and posttraumatic stress symptoms.

### Main findings and clinical implications

Thematic analysis revealed improvements in self-compassion, emotion regulation, self-image, and relational functioning. Participants attributed these changes to core elements of the MBT-TF framework. The structured and predictable group format—featuring fixed routines, visual schedules, and personalized tools—was described as essential for emotional safety and engagement. This predictability, combined with feeling seen and validated by peers, appeared to foster epistemic trust and openness.

Importantly, participants described—for the first time—a sense of belonging within a group, where they felt safe to express their authentic selves and experienced acceptance without judgment. The homogeneous neurodiverse composition of the group may have played a key role in this, offering a shared social context that reduced the cognitive and emotional demands of cross-neurotype interaction. This aligns with recent findings suggesting that autistic individuals often experience more effective social interaction and mutual understanding within peer groups composed of other autistic individuals (10). The shared neurotype may have facilitated a more intuitive grasp of each other’s internal states, enhancing the group’s collective capacity for mentalizing.

Despite initial concerns about group engagement due to participants’ neurodiverse profiles and histories of treatment dropout, attendance was exceptionally high, with only two absences over six months. This pattern indicates strong group cohesion and personal investment. We hypothesize that both the inherent qualities of MBT-TF and the specific structural adaptations—such as increased predictability, visual scaffolding, and personalized tools—enhanced emotional safety and epistemic trust, thereby supporting sustained engagement and therapeutic depth. Evidence for this included the complete absence of drop-out, high attendance rates, and participants’ active involvement in core therapeutic tasks, such as the co-construction of visual tools, use of structured reflections, and willingness to engage in trauma narrative work. Given participants documented difficulties maintaining participation in previous treatments, this stable engagement suggests that the adapted structure enabled deeper therapeutic work than had been possible in earlier interventions.

Participants were not only recipients of the adapted treatment but also active co-creators of its structure. Their input directly shaped several key modifications, including the replacement of the traditional “take-home message” with a structured self-report format and the integration of peer-generated questions during trauma narrative sessions. This collaborative process may itself have functioned as a meta-mentalizing intervention, reinforcing agency, ownership, and reflective functioning through shared decision-making.

These findings invite a broader conceptualization of therapeutic responsiveness—one that extends beyond moment-to-moment attunement to include structural and relational adaptations that align with neurodiverse processing styles. Rather than viewing autistic traits as barriers to group therapy, this study supports the notion that when relational contexts are appropriately adapted, individuals with ASD features can engage deeply and meaningfully in trauma-focused group work.

Psychoeducation and group dialogue helped participants gain distance from overwhelming emotional states and consider alternative perspectives, supporting emotion regulation and a more compassionate self-view. Feeling seen and validated by peers contributed to shifts in relational expectations and trust, aligning with MBT-TF’s aim to recalibrate trauma-related representations of self and others through shared mentalizing.

Quantitative findings supported these insights: three of four participants showed improvements across multiple domains. Although not all changes reached clinical significance, the convergence of data sources suggests meaningful progress and supports the feasibility of MBT-TF in this novel population.

This pilot study has several limitations that should be considered when interpreting the findings. First, the absence of a control group limits the ability to attribute observed changes specifically to the MBT-TF module, as naturalistic improvement or nonspecific therapeutic factors cannot be ruled out. Second, outcomes were based on self-report questionnaires, which are inherently susceptible to response bias, social desirability, and fluctuations in self-reflective capacity. Third, although the module was delivered as part of an existing adapted MBT-A program rather than as concurrent treatment, the broader therapeutic context may nevertheless have contributed to the observed changes. As a result, the specific effects of the MBT-TF component cannot be isolated with certainty.

## Conclusion and future directions

This pilot study provides initial support for the feasibility and relevance of MBT-TF in a marginalized and clinically complex population, including late adolescents with co-occurring C-PTSD, personality dysfunction, and ASD features. The intervention appears to engage individuals often considered difficult to treat, offering a structured, relationally focused approach that fosters epistemic trust and supports therapeutic change.

Future research should replicate these findings in larger and more diverse samples, using longitudinal designs and observer-rated measures to assess sustained impact and mechanisms of change. Further refinement of the MBT-TF manual to better accommodate neurodiverse populations is recommended to enhance accessibility and clinical utility.

## Data Availability

The raw data supporting the conclusions of this article will be made available by the authors, without undue reservation.
