# Supplementary material for: Mentalizing in the margins: a pilot study of group-based MBT-TF for underserved neurodiverse late adolescents
Source: Front Psychiatry. 2026 Apr 28;17:1743200. doi: 10.3389/fpsyt.2026.1743200 (PMC13162092; doi:10.3389/fpsyt.2026.1743200)
Supplement: Supplementary file 1 [file SupplementaryFile1.docx]

|  |  |  | |  |  |
| --- | --- | --- | --- | --- | --- |
|  | Client ID |  | |  |  |
|  | 1 | 2 | | 3 | 4 |
| Age | 18 | 18 | | 19 | 23 |
| Current diagnosis | Autism spectrum disorder, other specified personality disorder, depressive disorder | Other specified trauma- and stressorrelated disorder, autism spectrum disorder, persistent depressive disorder, gender dysphoria | | Post traumatic stress disorder, avoidant personality disorder, other specified attention deficit/hyperactivity disorder, avoidant/restrictive food intake disorder** | Other specified trauma- and stressorrelated disorder, borderline personality disorder, anorexia nervosa, attention deficit/hyperactivity disorder inattentive presentation, tourette syndrome** |
| Additional sessions during module | Individual treatment, family therapy | None | | None | Individual treatment, family therapy |
| CTQ pre | 66 | 48 | | 65 | 72 |
| CTQ post | 72 | 40 | | 59 | 93 |
| CTQ Δ | +6 | -8 | | -6 | +21 |
| CTQ RCI | +0.86 | -1.14 | | -0.86 | +3.00* |
| RFQ pre | 31 | 37 | | 26 | - |
| RFQ post | 42 | 33 | | 41 | 42 |
| RFQ Δ | +11 | -4 | | +15 | - |
| RFQ RCI | +1.57 | -0.57 | | +2.14 | - |
| IIP pre | - | 15.38 | | 20.64 | 14.13 |
| IIP post | - | 14.66 | | 12.51 | 11.40 |
| IIP Δ | - | -0.72 | | -8.13 | -2.73 |
| IIP RCI | - | -0.36 | | -4.07* | -1.37 |
| DES pre | - | 37.21 | | 81.29 | 72.32 |
| DES post | - | 45.54 | | 42.32 | - |
| DES Δ | - | +8.33 | | -38.97 | - |
| DES RCI | - | +1.11 | | -5.20* | - |
| PCL pre | - | 42 | | 65 | 58 |
| PCL post | - | 47 | | 44 | 46 |
| PCL Δ | - | +5 | | -21 | -12 |
| PCL RCI | - | +0.71 | | -3.00* | 1.71 |
| *Note. *p<.05; **there was a broad and persistent pattern of autistic characteristics* | | |  |  |  |
|  | |  | |  |  |

Appendix 1. Descriptives and pre and post scores of all participants
